# Maintenance Electroconvulsive Therapy in a Patient with Treatment-Resistant Paranoid Schizophrenia and Comorbid Epilepsy

**DOI:** 10.1155/2012/374752

**Published:** 2012-07-03

**Authors:** Beppe Micallef-Trigona, Joseph Spiteri

**Affiliations:** Department of Psychiatry, Mount Carmel Hospital, Attard, ATD 9033, Malta

## Abstract

The treatment of choice for acute schizophrenia is antipsychotic drug treatment and electroconvulsive therapy (ECT) and should only be considered as an option for treatment-resistant schizophrenia, where treatment with clozapine has already proven ineffective or intolerable. The use of ECT as a maintenance treatment for patients with schizophrenia and comorbid epilepsy is uncommon as scant evidence exists to support this. We describe a patient with a serious case of paranoid schizophrenia and comorbid epilepsy who had not responded to typical and atypical antipsychotic medication, but responded remarkably to acute ECT and required maintenance ECT to sustain a positive therapeutic response.

## 1. Introduction

ECT has been recognized as an effective treatment option in the treatment of acute schizophrenia [1]. However it is usually limited to use as a fourth line option, that is, an option for treatment-resistant schizophrenia after treatment with two different antipsychotics as well as clozapine has proved to be ineffective or the patient has been unable to tolerate such treatment [2]. Nonetheless, even in these cases, the use of ECT has been questioned and in fact, NICE guidelines specify that “the current state of the evidence does not allow the general use of ECT in the management of schizophrenia to be recommended” [3]. As a result, ECT has had scarce use as a form of maintenance therapy, after the acute phase of schizophrenia, despite reports of patients showing improvement without relapse/recurrence [4–6]. To add to this predicament, the use of ECT in patients who also suffer from epilepsy is uncommon as there is little published data available [7] and no guidelines for safe and effective use of ECT in such patients.

## 2. Case Report

Mrs F was a 50-year-old female, admitted to our psychiatric hospital in August of 2010 with a two-month history of auditory hallucinations, paranoid delusions, and chaotic and aggressive behaviour. She had been originally diagnosed with paranoid schizophrenia (F20.0) according to the criteria of the International Classification of Diseases (ICD-9 [8], after admission to our psychiatric hospital in February 1985, but since discharge had remained relatively stable in the community for over two decades. Four months before her readmission she was on risperidone (2 mg/day) and chlorpromazine (25 mg/day) together with her medical treatment; she also suffered from asthma, diabetes mellitus, hypercholesterolemia, and epilepsy. The latter had been diagnosed 6 years previously, following neurologist consultation, due to 3 episodes of (witnessed) tonic-clonic seizures, with loss of consciousness and clear postictal phase. The patient was commenced on phenytoin sodium (100 mg/day) and remained well-controlled since. 

Her relapse was thought to be due to incompliance and therefore her treatment was restarted. However her symptoms did not improve and her dose of risperidone was increased (to 6 mg/day). Her chaotic and aggressive behaviour persisted as did her delusions; these were mood-neutral, nonbizarre, and paranoid delusions which revolved around her daughters and the staff of the psychiatric ward, who she accused of trying to poison her. She in fact began to refuse oral treatment, and after receiving haloperidol intramuscularly (up to 30 mg/day) over a period of two days, she was then prescribed zuclopenthixol decanoate depot (400 mg/2 weeks). This did not improve her mental state and over the following months she received a trial of a number of different psychotropics: quitiapine (up to 800 mg/day), flupentixol (up to 3 mg/day), trifluoperazine (up to 20 mg/day), sodium valproate (up to 1000 mg/day), and fluphenazine depot (75 mg/4 weeks). The patient however would frequently develop debilitating extra pyramidal side effects and although her aggressive outbursts had decreased, she remained disorganised and steadfast in her delusions. She would enter most wards wounds clutching a heap of random papers which she would hand over to me whilst saying “here are the donations I have collected”. 

She was started on clozapine after having been admitted for 10 months but developed neutropenia after 3 weeks. 6 sessions of modified bilateral ECT were therefore prescribed, at a frequency of 2 × week. By the 5th session there was little clinical improvement but following her 6th ECT, she walked into the ward round room and one could tell that a change had taken place. She no longer handed over her routine “donations” and informed us that she was now feeling well. We enquired into her delusions and she told us that it was all “just a story she had made up.” The change was marked and was confirmed by a reduction in score of the 18-item Brief Psychiatric Rating Scale (BPRS) [9] from 68 before her 1st ECT to 38 after her 6th. 

A decision was made to continue the administration of bilateral modified ECT once weekly. By the 8th administration she was able to go on leave with her daughters and began attending ECT as an out-patient. Her psychiatric treatment was tailed down to include haloperidol (17.5 mg/day), quitiapine (100 mg/day), and sodium valproate (500 mg/day—guided by blood level monitoring). After the 11th administration prescription was changed to right unilateral modified ECT. She remained well and after the 15th administration, ECT was tailed down to fortnightly. However, before her 17th session was due, the patient began complaining of insomnia and hinting that her daughter was confusing her medications. It was therefore decided to continue right unilateral modified ECT once weekly. She immediately improved and no further deterioration was noted. Up until now the patient has been administered a total of 32 sessions of ECT and remains stable on leave from the hospital, having seen a reduction in her BPRS score to 23. Her MMSE score is measured twice monthly and has not shown any deterioration. 

## 3. Discussion

As had been described previously in another case report [6] we observed a patient with treatment-resistant paranoid schizophrenia that showed marked response to ECT, which persisted on maintenance administration. In our case the patient had not withstood clozapine due to the emergence of neutropenia. In combination with ECT, our patient was prescribed the oral antipsychotics haloperidol and quitiapine, which, as demonstrated by a number of studies [10, 11], is safe and efficacious for treatment-resistant schizophrenia.

Our case report also demonstrated that ECT may be beneficial and safe in patients with treatment-resistant schizophrenia who also suffer from concurrent epilepsy. According to Lunde et al. [7] there is limited published data to guide the clinician about safe and effective use of ECT in epileptic patients who suffer from psychiatric disorders, but they conclude that most epileptic patients can be treated safely with ECT without dose adjustment in antiepileptic medications. Coffey et al. [12] discovered that seizure threshold increased by approximately 47% on average in the patients taking part in their study. However, throughout her administration of ECT, our patient remained well controlled and seizure-free outside ECT suite, and as Lunde et al. had proposed, no adjustment in her phenytoin sodium dose was needed.

Due to the fact that the patient is now on what can be termed “long-term” ECT treatment, one must bear in mind possible adverse effects, especially memory impairment. Despite the controversy surrounding this issue [13], it would be wise to keep a close watch on such adverse effects through frequent monitoring of cognitive status.

Further study is definitely required in order to create and standardize guidelines for prescription, continuation, and maintenance ECT for patients with treatment-resistant schizophrenia.

## 4. Conclusion

This case report suggests that pharmacological treatment resistant paranoid schizophrenia can respond to continuation and maintenance of ECT and is safe for patients with comorbid epilepsy.
